# Supplementary material for: Host PIK3C3 promotes Shigella flexneri spread from cell to cell through vacuole formation
Source: PLoS Pathog. 2025 May 16;21(5):e1012707. doi: 10.1371/journal.ppat.1012707 (PMC12165337; doi:10.1371/journal.ppat.1012707)
Supplement: S1 Table — Hits obtained in three biological replicates (R1-3) showing infection foci size and corresponding Z-Scores. Hits were selected on the basis of a decrease of infection foci size by at least 2 standard deviations (stdev WT), i.e., Z-Score < -2, with respect to the average (avg) foci size in control wells (avg WT) containing DMSO only. (PDF) [file ppat.1012707.s001.PDF]

	Replicate	R1		R2		R3	
	avg WT	3569		3290		3684	
	stdev WT	257		324		228	
Compound	Target	Foci size	Z-Score	Foci size	Z-Score	Foci size	Z-Score
KU-60019	ATM	2280.5	-5.0	1835.6	-4.5	2635.2	-4.6
A-674563	Akt, CDK, PKA	2916.1	-2.5	1933.4	-4.2	2361.8	-5.8
GSK2126458	PI3K, mTOR	1186.5	-9.3	1421.4	-5.8	2022.6	-7.3
GSK2292767	PI3K	2699.0	-3.4	2103.5	-3.7	2841.5	-3.7
VPS34-IN1	PI3K	1792.6	-6.9	1951.4	-4.1	2012.1	-7.3
AZD5438	CDK	2649.8	-3.6	2194.1	-3.4	2416.7	-5.6
YM201636	PI3K	2886.1	-2.7	2421.7	-2.7	2900.3	-3.4
