# Supplementary figures and images for: Host PIK3C3 promotes Shigella flexneri spread from cell to cell through vacuole formation
Source: PLoS Pathog. 2025 May 16;21(5):e1012707. doi: 10.1371/journal.ppat.1012707 (PMC12165337; doi:10.1371/journal.ppat.1012707)

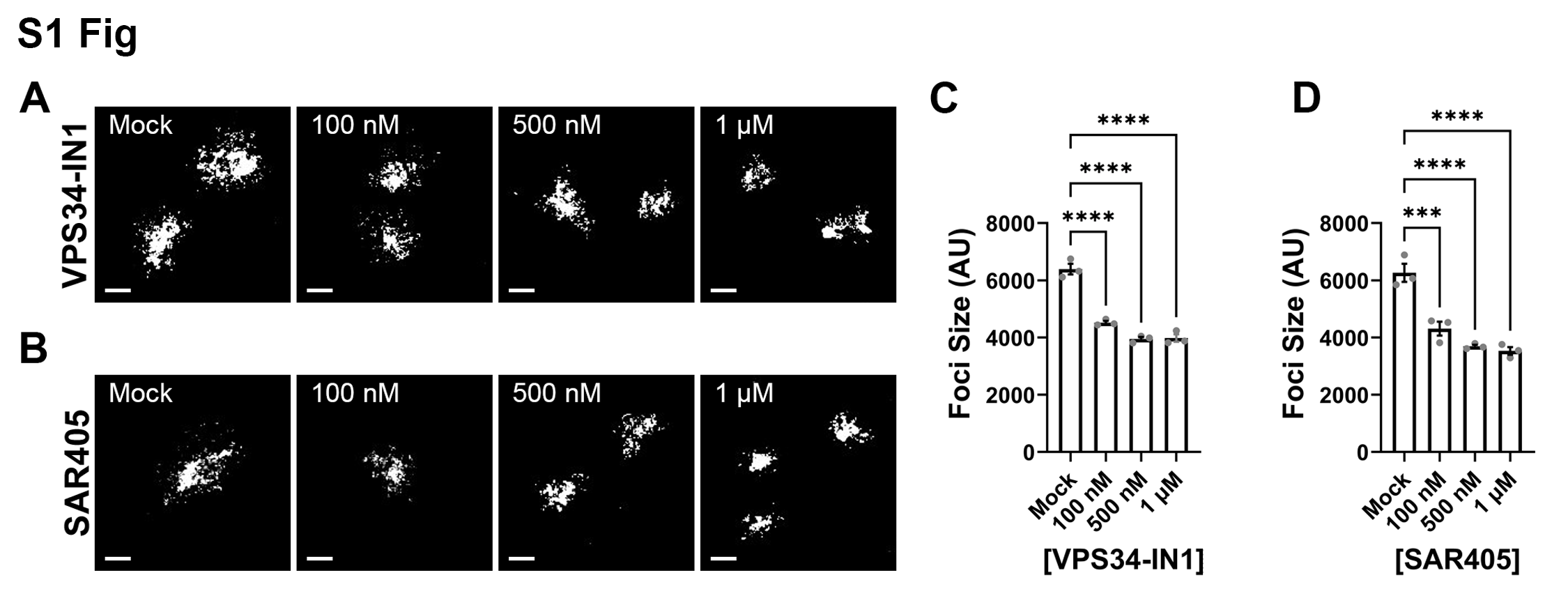

Supplement: S1 Fig — (A-B) Representative images showing infection foci formed by CFP-expressing S. flexneri in Caco-2 cells 8 hours post-infection in presence of the specific PIK3C3 inhibitor VPS34-IN1 (A) or SAR405 (B). Scale bar, 50 μm. (C-D) Quantification of foci size (area) in arbitrary units 8 hours post-infection in presence of VPS34-IN1 (C), or SAR405 (D). Each dot represents the average of one biological replicate. At least 50 infection foci were analyzed in each of three independent biological replicates. Error bars represent standard error of the mean. Statistical analysis, one-way ANOVA with Dunnett’s multiple comparisons test; ns, not significant; ***, p < 0.001; ****, p < 0.0001. (TIF) [file ppat.1012707.s003.tif]

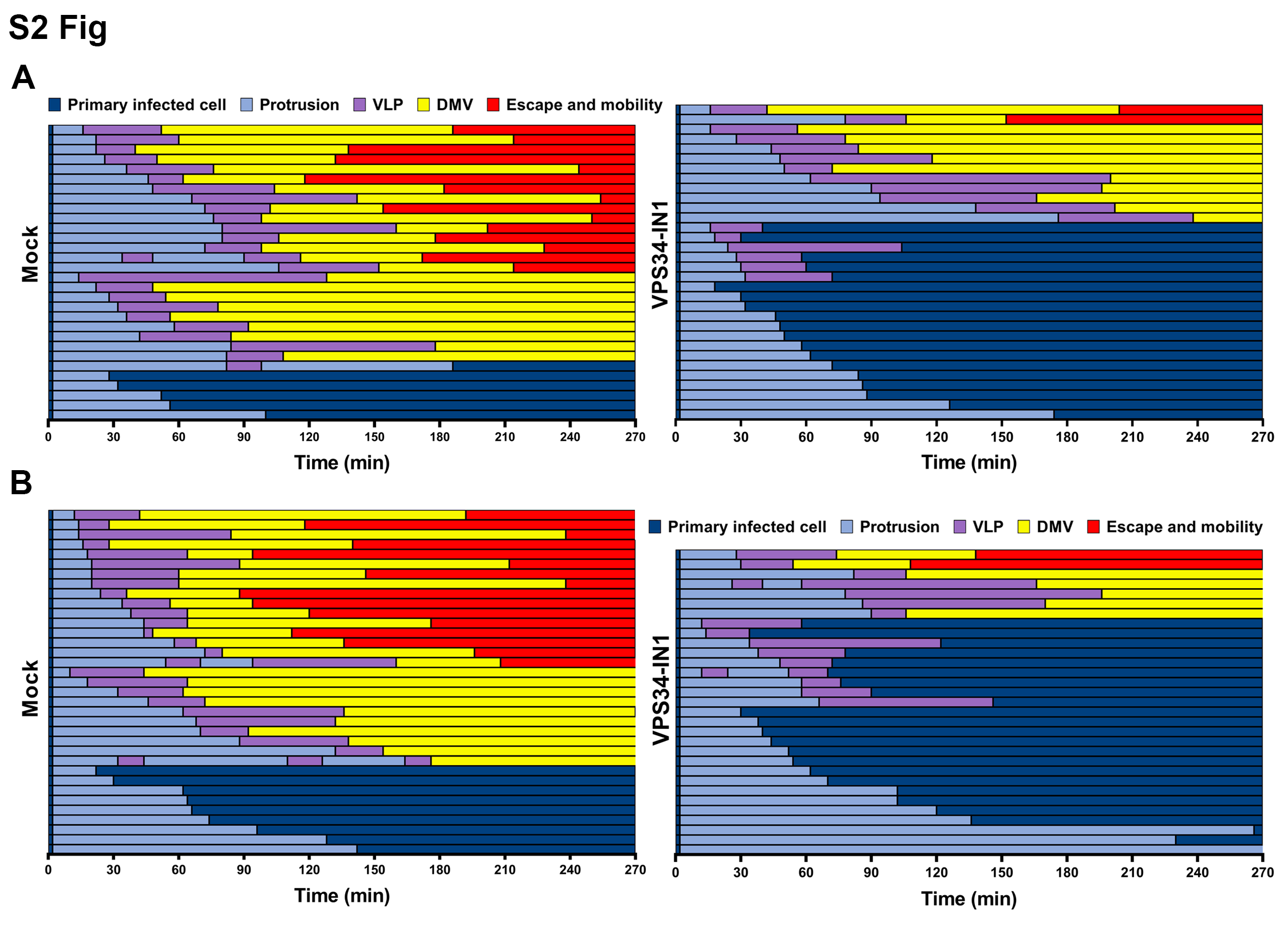

Supplement: S2 Fig — (A-B) Representative tracking analysis of second (A) and third (B) biological replicates using live-fluorescence confocal microscopy of CFP-expressing S. flexneri during cell-to-cell spread in the absence (left panel) or presence (right panel) of VPS34-IN1 (500 nM). Each bar represents the tracking of a single bacterium over 4h30. Color code: dark blue, primarily infected cells; light blue, protrusion; purple, vacuole-like protrusion (VLP); yellow, double membrane vacuole (DMV) and red, escape and actin-based motility in the cytosol of adjacent cells. (TIF) [file ppat.1012707.s004.tif]

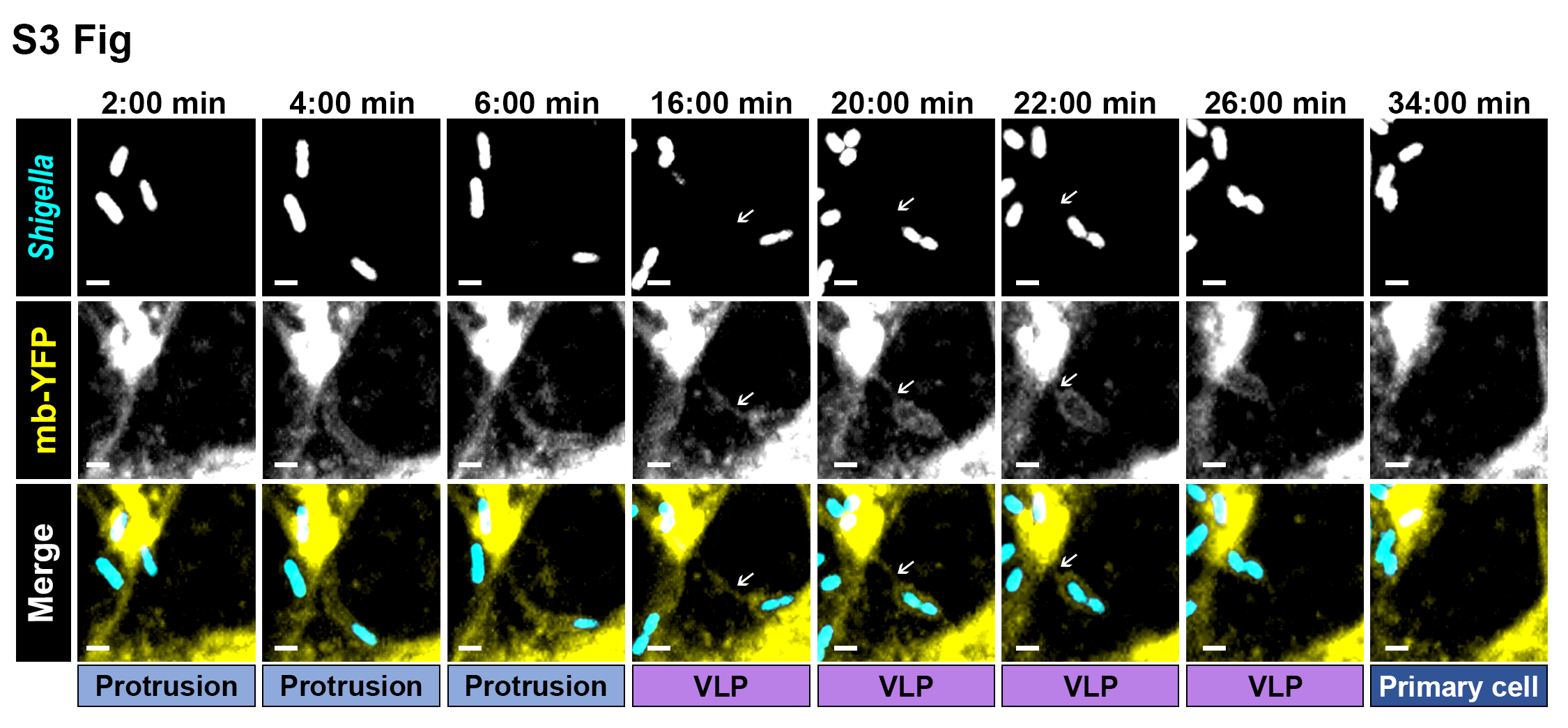

Supplement: S3 Fig — Live-fluorescence confocal microscopy images of membrane YFP-expressing HT-29 cells infected with CFP-expressing S. flexneri in presence of VPS34-IN1 (500 nM). The tracked bacterium forms a protrusion that successfully resolves into a VLP, but does not resolve into a DMV. The VLP subsequently retracts back to the primary infected cell. The arrows indicate the membranous tether formed during the VLP stage. Scale bar, 2 μm. (TIF) [file ppat.1012707.s005.tif]

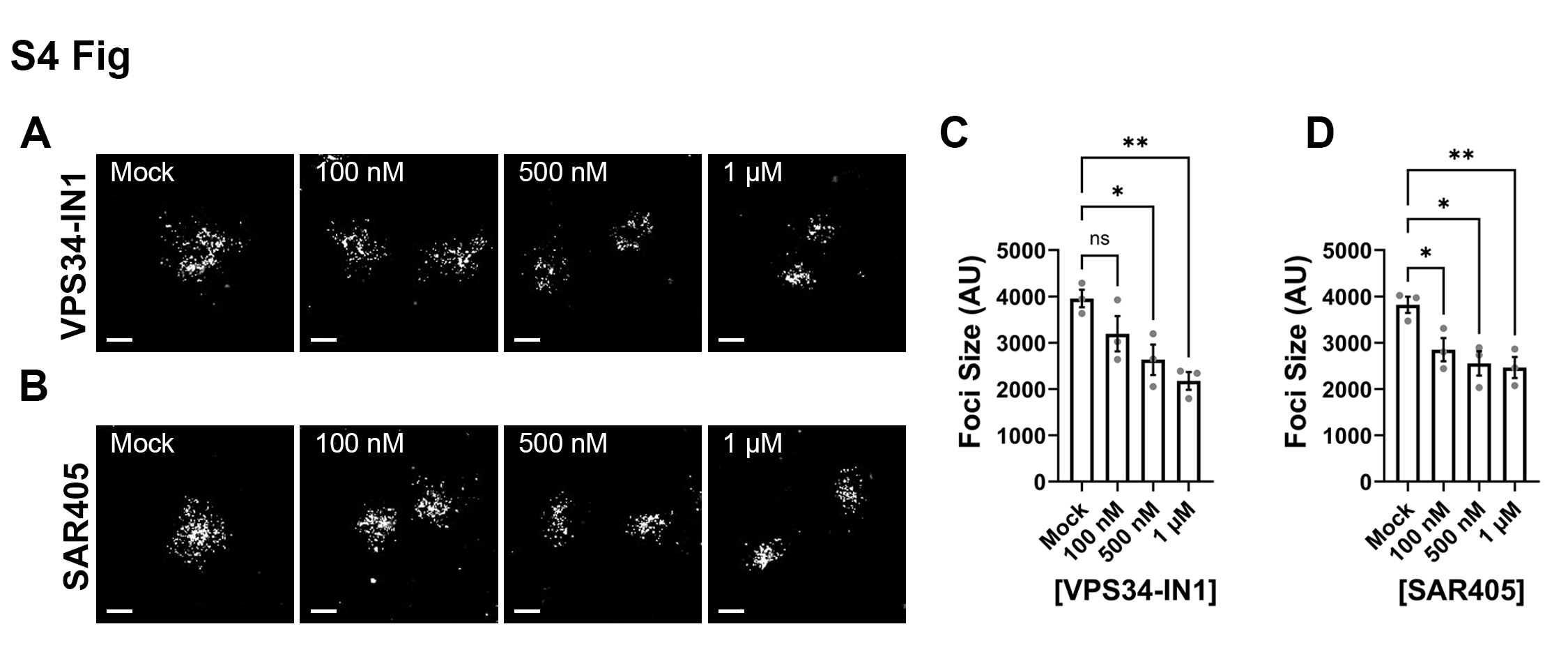

Supplement: S4 Fig — (A-B) Representative images showing infection foci formed by GFP-expressing L. monocytogenes in Caco-2 cells 8 hours post-infection in presence of the specific PIK3C3 inhibitor VPS34-IN1 (A) or SAR405 (B). Scale bar, 50 μm. (C-D) Quantification of foci size (area) in arbitrary units 8 hours post-infection in presence of VPS34-IN1 (C), or SAR405 (D). Each dot represents the average of one biological replicate. At least 50 infection foci were analyzed in each of three independent biological replicates. Error bars represent standard error of the mean. Statistical analysis, one-way ANOVA with Dunnett’s multiple comparisons test; ns, not significant; *, p < 0.05; **, p < 0.01. (TIF) [file ppat.1012707.s006.tif]

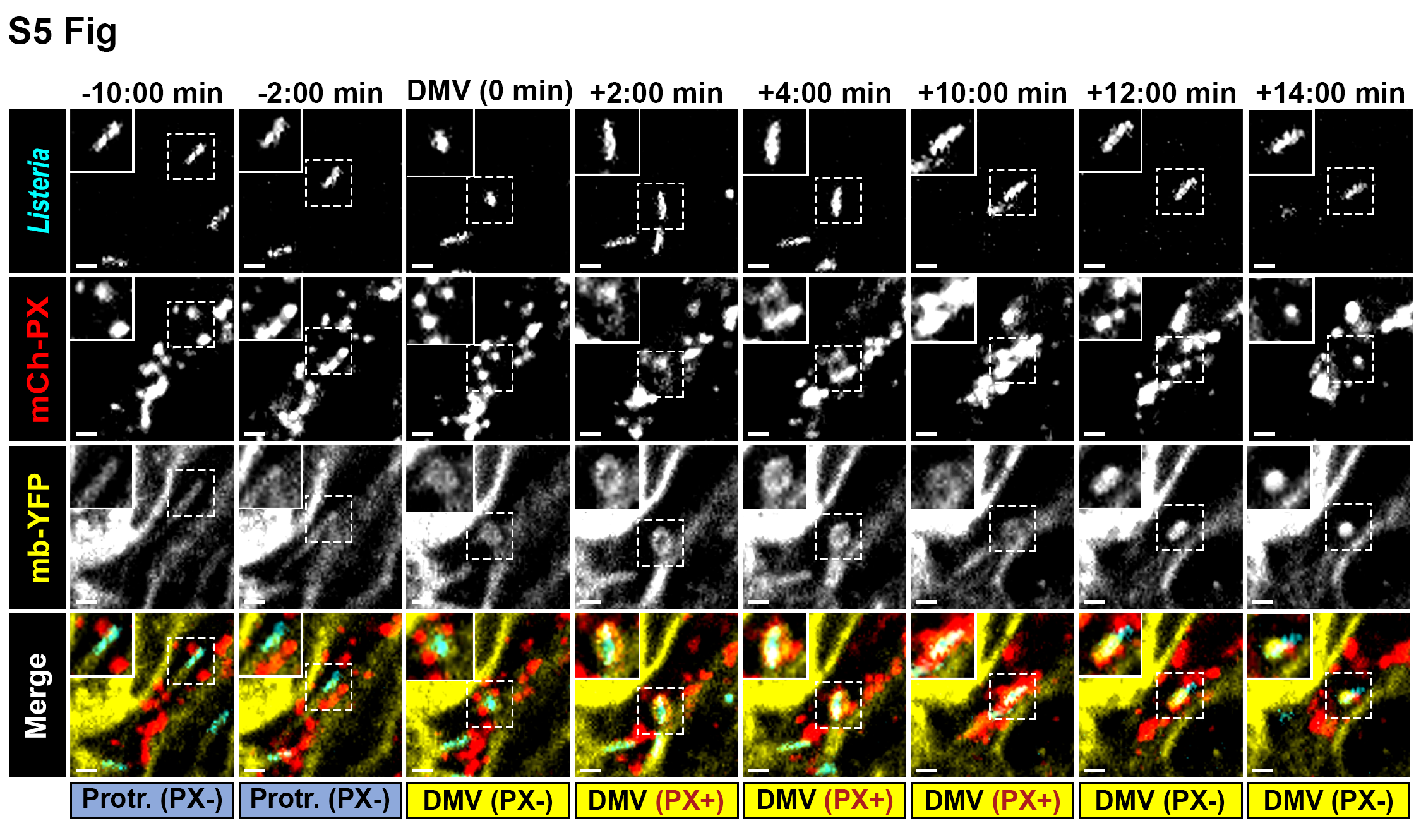

Supplement: S5 Fig — Live-fluorescence confocal microscopy images of membrane YFP-expressing HT-29 cells infected with CFP-expressing L. monocytogenes spreading from a mCherry-PX non-expressing cell into a mCherry-PX expressing cell. Unlike S. flexneri that forms VPLs, the frame before DMV formation shows a protrusion in cells infected with L. monocytogenes. Scale bar, 2 μm. (TIF) [file ppat.1012707.s007.tif]

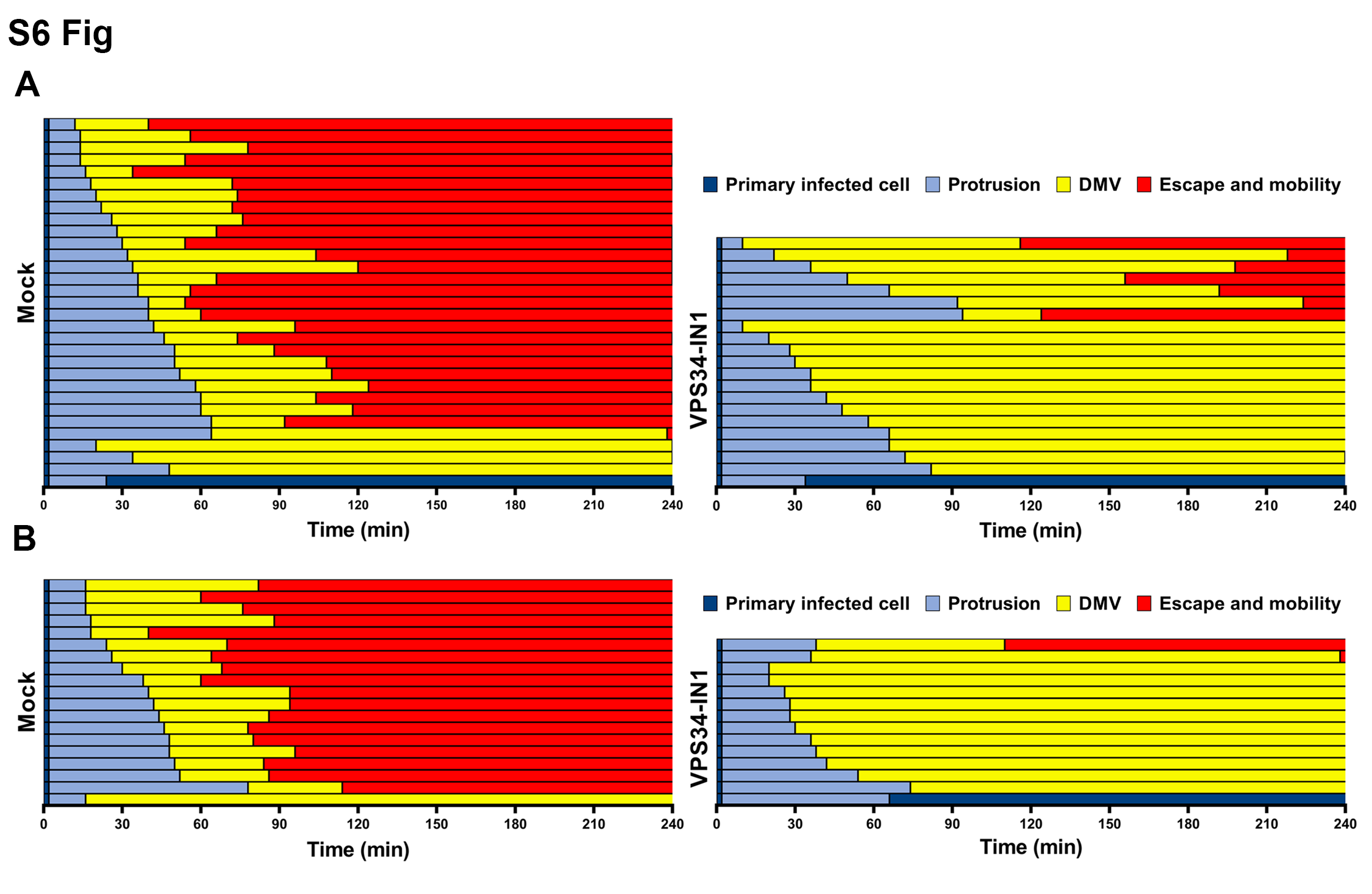

Supplement: S6 Fig — (A-B) Representative tracking analysis of the second (A) and third (B) biological replicates using live-fluorescence confocal microscopy of CFP-expressing L. monocytogenes during cell-to-cell spread in absence (left panel) or presence (right panel) of VPS34-IN1 (500 nM). Each bar represents the tracking of a single bacterium over 4h. Color code: dark blue, primarily infected cells; light blue; yellow, double membrane vacuole (DMV) and red, escape and mobility in the cytosol of adjacent cells. (TIF) [file ppat.1012707.s008.tif]

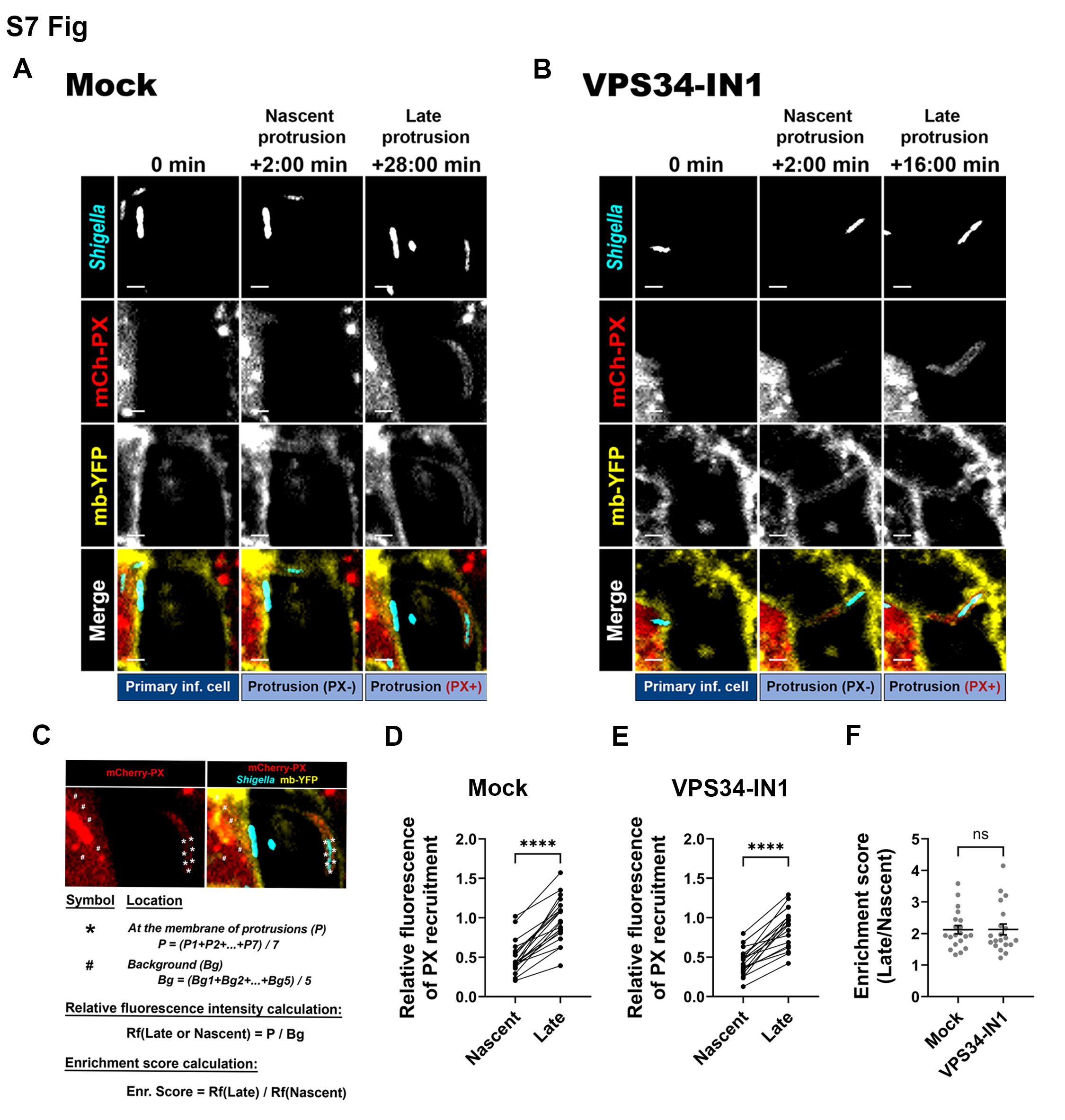

Supplement: S7 Fig — (A-B) Representative images of membrane YFP-expressing cells infected with CFP-expressing S. flexneri dissemination from PX-mCherry expressing cells to PX-mCherry non-expressing cells in absence (A, Mock) or presence of VPS34-IN1 at 500 nM (B). PX-mCherry specifically binds PtdIns(3)P. Scale bar, 2 μm. (C) Representative example of relative fluorescence intensity and enrichment score calculation. (D-E) Graphs representing how mCherry-PX relative fluorescence intensity change between nascent protrusion membrane and late protrusion membrane in absence (D) or presence of VPS34-IN1 at 500 nM (E). 20 bacteria were tracked per condition. Statistical analysis, paired t-tests were performed; ns, not significant; ****, p < 0.0001. (F) Graphs representing the enrichment score calculated based on the relative fluorescence intensity of the late and nascent protrusion as described in panel C in presence or absence of VPS34-IN1. Statistical analysis, unpaired t-tests were performed, ns; not significant. (TIF) [file ppat.1012707.s009.tif]

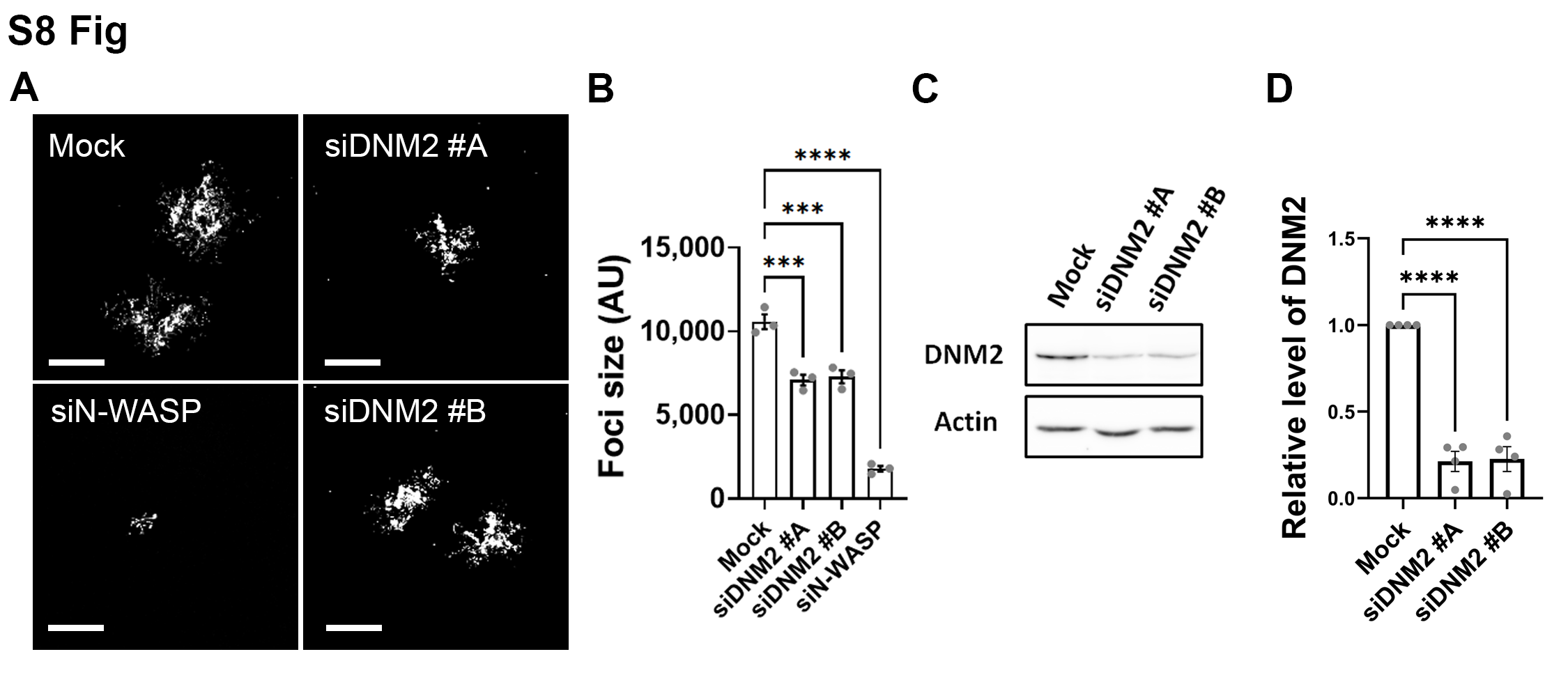

Supplement: S8 Fig — (A) Representative images showing infection foci formed in HT-29 cells treated with two siRNA duplexes targeting DNM2 16 hours post-infection. Scale bar, 100 μm. (B) Quantification of foci size (area) as shown in (A). Three independent biological replicates were performed and at least 50 infection foci were analyzed per condition. Each dot represents the average of one independent experiment and error bars represent the standard deviation of the mean. Statistical analysis, one-way ANOVA with Dunnett’s multiple comparisons test; ns, not significant; *, p < 0.05; **, p < 0.005; ***, p < 0.001; ****, p < 0.0001. (C) Western blot showing knockdown efficiency of two siRNA duplexes targeting DNM2. (D) Quantification of the knockdown efficiency in three biological replicates. DNM2 signals were normalized to the corresponding actin signals and knockdown efficiency of siRNA duplexes was calculated relative to mock treated cells. Statistical analysis, one-way ANOVA with Dunnett’s multiple comparisons test; ns, not significant; ****, p < 0.0001. (TIF) [file ppat.1012707.s010.tif]
